# Death due to acute Chagas -related myocarditis in a child: a case report

**DOI:** 10.1590/0037-8682-0406-2019

**Published:** 2020-04-22

**Authors:** Theresa Cristina Cardoso da Silva, Karina Balestreiro Silva, Clemilda Soares Marques, Janaína Aparecida Schineider Casotti, Eveline de Fátima Almeida Fonseca Eduardo, Jane Sant’ana Castello, Maria Augusta Dario, Juliana Rodrigues Tovar Garbin, Sandra Fagundes Moreira-Silva

**Affiliations:** 1 Secretaria de Saúde do Estado do Espírito Santo, Núcleo Especial de Vigilância Epidemiológica, Vitória, ES, Brasil.; 2 Hospital Infantil Nossa Senhora da Glória, Departamento de Doenças Infecciosas, Vitória, ES, Brasil.; 3 Secretaria de Saúde do Estado do Espírito Santo, Centro de Informações Estratégicas em Vigilância em Saúde, Vitória, ES, Brasil.; 4 Fundação Oswaldo Cruz, Instituto Oswaldo Cruz, Laboratório de Biologia de Tripanosomatídeos, Manguinhos, RJ, Brasil.

**Keywords:** Chagas disease, Chagas-related cardiomyopathy, Trypanosoma cruzi, Child

## Abstract

This is a case report about the only confirmed death in the State of Espírito Santo due to acute Chagas-related myocarditis in a 2-year-old child living in the rural area of Guarapari. He presented with fever, abdominal pain, headache, and vomiting, resulting in death 21 days after the presentation of symptoms. Amastigote forms were observed in the myocardial fibers in histological examination. The boy’s mother had reported finding “kissing bugs” in the child’s hand. This case highlights the need to include Chagas disease in the differential diagnosis in health care to provide early treatment and avoid death in affected individuals.

## INTRODUCTION

Chagas disease, which is also referred to as American trypanosomiasis and was described in 1909 by Carlos Chagas, is caused by the flagellate protozoan *Trypanosoma cruzi*, whose vectors are triatomine bugs, commonly known as kissing bugs^1^.

More than one hundred years after its description, it is still a major public health problem, specifically in Latin America, causing disability in infected individuals, with more than 10,000 deaths annually^2^. The World Health Organization estimates that 8 million people have been infected with *T. cruzi* worldwide^2^.

Chronic cases, which have also been observed in previous decades, resulting in vector-borne infection are currently predominant in Brazil^3^. Between 2000 and 2013, it was observed that oral transmission was the most frequent (68.9%) cause, followed by vector transmission (6.4%)^3^.

In rural areas in the State of Espírito Santo (ES), home invasion by infected triatomine insects, mainly the *Triatoma vitticeps* species, is common, with high rates of *T. cruzi* infection (86.2%)^4^. However, according to studies conducted in the state, despite the high percentage of positivity found in the triatomine bugs in the region, low endemicity of the disease was observed with few autochthonous cases recorded, suggesting that this species is possibly not a good vector of the disease^5^.

This study aims to report the death of a child with acute Chagas-related myocarditis in a state children’s hospital in ES in 2012, the only confirmed death due to acute Chagas disease (ACD) in ES. Thus, the present study highlights the importance of establishing the diagnosis and early treatment for ACD to achieve favorable outcomes.

The study was authorized by the State Department of Health of ES (protocol number: 82551332), and it was approved by the research ethics committee of the Superior School of Sciences of *Santa Casa de Misericórdia de Vitória* through the Brazil Platform (opinion number: 3,093,203).

## CASE REPORT

The patient identified as LFBL was 2 years and 10 months old and was a white male from Rio da Prata, a rural area of the Municipality of Guarapari. He had no history of blood transfusion. He was previously healthy, but on February 13, 2012, he presented with fever, abdominal pain, headache, and vomiting. After 3 days, he developed a generalized skin rash.

He initially received symptomatic relief medications on February 16, 2012 and underwent a hemogram. According to the hemogram result, the patient presented with anemia and thrombocytopenia.

Considering the clinical condition that showed acute febrile illness and the hemogram result, the patient was suspected with dengue fever; hence, a specific serology collection was scheduled. New hemograms were performed showing normal leukogram and platelet counts. On February 20, 2012, the patient was treated at the health unit because of a declined general condition and low-grade fever, and complementary tests revealed the following: hemoglobin, 9.9 g/dl; hematocrit, 29.2%; leukocytes, 4,500/mm^3^; and platelet count, 75,000/mm³.

On February 24, 2012, the child still had a decline of his general condition with a low-grade fever. The hemogram revealed leukocytosis, lymphocytosis, and thrombocytosis. On March 1, 2012, the child was treated again at the health unit and was subsequently discharged.

On April 3, 2012, 20 days after illness onset, with a worsening clinical condition, the child returned to the Guarapari emergency care unit. According to the test results, he was dehydrated with cutaneous pallor, eupneic, and afebrile. Hence, he received symptomatic relief medications and was discharged following the diagnosis of acute gastroenteritis. Later that day, after having fainted, his family took him back to the emergency care unit. Upon admission, his blood glucose level was 53 mg/dl, and he experienced dyspnea and abdominal distension without diuresis. His hemogram revealed the following: hemoglobin, 8.6 g/dl; hematocrit, 28.2%; leukocytes, 13,100/mm^3^; and platelet count, 304,000/mm^3^. Hence, the child had to be transferred to a hospital.

Considering the patient’s significantly serious general condition, he was admitted on May 3, 2012 in the emergency department of the benchmark hospital (*Hospital Infantil Nossa Senhora da Glória*). He required oxygen because he experienced difficulty in breathing. Moreover, he was dehydrated with cold extremities due to slow blood perfusion and was gently groaning and tachypneic with bleeding like “coffee grounds” (shown) in the gastric tube without diuresis. An X-ray examination of the thorax and abdomen revealed diffuse pulmonary infiltrates with right pleural effusion and gastric distension. The laboratory tests revealed the following: capillary blood glucose, 52 mg/dl; hemoglobin, 13.1 g/dl; hematocrit, 41.8%; leukocytes, 15,600/mm^3^; platelet count, 346,000/mm^3^; urea, 157; creatinine, 21.9; glutamic-oxalacetic transaminase, 181; glutamic-pyruvic transaminase, 72; albumin, 2.19; prothrombin time, 27.6%; and activated partial thromboplastin time, 63%.

**FIGURE 1:**
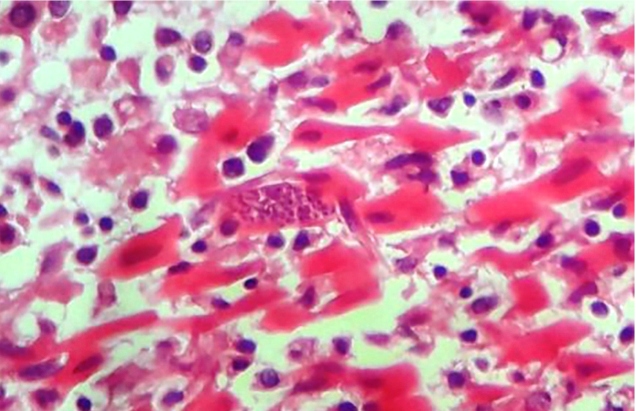
Amastigote forms in the myocardial fibers in the histological examination of the patient’s heart.

The condition evolved unfavorably after a few hours, and the patient experienced cardiorespiratory arrest that was not responsive to the procedures performed. He subsequently died, and his body was sent for necropsy. The macroscopic findings were as follows: pallid myocardium, thickened right ventricle, epicardium with petechiae, bilateral hydrothorax, serous hydroperitoneum, cerebral edema, and hemorrhage in the gastric mucosa sheet. In line with these findings, the following death certificate was issued: cerebral edema, pulmonary collapse, bilateral hydrothorax, ascites, and probable hemorrhagic dengue fever. Later on, a histopathological examination revealed amastigote forms in the myocardial sections (Figure 1), confirming death due to Chagas-related myocarditis.

Dengue immunoglobulin M/enzyme-linked immunosorbent assay serology, the main differential diagnosis established in this patient, which was collected on the 11th day of illness, was negative, ruling out dengue.

**FIGURE 2:**
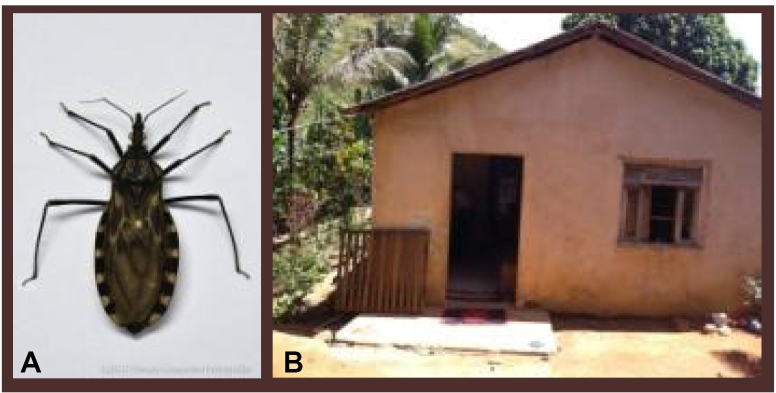
**(A)** *Triatoma vitticeps*. **(B)** The patient’s residence.

The child’s house was located in a rural area, which was hard to reach with a narrow dirt road. It was a simple masonry house with cracks in the roof and windows and a significantly large yard with fruit trees, domestic animals, and wood scraps and rubbish. According to his mother, she had been bitten by kissing bugs, which always appeared at night in the house. She had also found a triatomine bug in the child’s hand, suggesting the bug’s oral transmission. *Triatoma vitticeps* was found in the child’s home (Figure 2).

Approximately 300 meters from this house resides the child’s paternal grandfather and uncle, who own a sugar cane plantation and a cane grinder. The child was able to consume sugar cane juice approximately 40 days before he presented the initial symptoms. Direct parasitological examinations and serologies of 12 individuals living in the area were performed, and all the results were negative. As there were no other similar cases in the family, the hypothesis of oral transmission by contaminated foods has been ruled out.

The Nucleus of Entomology and Malacology of Espírito Santo (NEMES) reported that the existence of *T. vitticeps* in the Municipality of Guarapari was already observed due to specific studies, confirming 61.5% positivity for the parasite *T. cruzi* in the specimens examined in 2010 and 100% positivity in the specimens examined in 2011.

## DISCUSSION

The case described is the only confirmed death due to myocarditis caused by ACD in a child in the State of ES confirmed by a histopathological examination and polymerase chain reaction of mixed infection by different *T. cruzi* genotypes, besides the presence of *Trypanosoma dionisii* ^6^.

ACD was described by Carlos Chagas in Lassance in the State of Minas Gerais as an infection that occurs predominantly in children in their first decade of life^1^. A total of 233 cases of the ACD from Pará, Amapá, and Maranhão were observed between 1988 and 2005. Among the most frequent clinical manifestations is myocarditis with occurrence in 39.9% of the cases. Thirteen (5.6%) patients died, with ten (76.9%) of them due to cardiovascular involvement^7^.

An acute phase that can be symptomatic or asymptomatic has been shown, and it can progress to the chronic phase^8^. In the acute phase, fever, myalgia, headache, prostration, rash, and signs and symptoms of cardiac involvement such as lower limb edema, tachycardia, and hepatosplenomegaly may occur^1^ ^-^ ^8^ ^,^ ^9^.

More severe cases with myocardiopathy and meningoencephalitis are more frequent in children^1^ ^-^ ^10^ ^,^ ^11^ due to infection by oral transmission^4^ ^-^ ^11^. Approximately 5%-10% of symptomatic patients die^8^, whereas the other 90% experience the resolution of symptoms spontaneously or with specific treatment^8^.

Healthcare professionals’ insufficient suspicion on the disease and delay in treatment worsen the prognosis, which can lead to death, as in the case described.

Triatomine bugs associated with a high degree of *T. cruzi* infection invading the residences remains unchanged in the rural areas of the Municipality of Guarapari, and the risk of new cases is significantly high^5^.

This report highlights the importance of adequate control of the disease, and healthcare teams, with an emphasis on the primary care teams, should incorporate surveillance actions in their work process, specifically in rural areas where the homes are frequently infected by triatomine bugs, which may lead to disease outbreak.

Programs aimed at educating the individuals residing in these areas, healthcare professionals, and local medical teams in determining the stages of the disease, from identifying the risks and forms of transmission to symptom onset to determining the kissing bugs and treating ACD, are required. With sufficient knowledge regarding the ACD status in each region, a better control of ACD is established.
